# The complete chloroplast genome sequence of *Taxus yunnanensis*

**DOI:** 10.1080/23802359.2020.1788442

**Published:** 2020-07-11

**Authors:** Yunfen Geng, Yunqin Li, Xiaolong Yuan, Mei Hua, Yi Wang, Jinfeng Zhang

**Affiliations:** Laboratory of Forest Plant Cultivation and Utilization, Yunnan Academy of Forestry & Grassland Science and The Key Laboratory of Rare and Endangered Forest Plants of State Forestry Administration, Kunming, Yunnan, People's Republic of China

**Keywords:** *Taxus yunnanensis*, chloroplast, Illumina sequencing, phylogenetic analysis

## Abstract

The first complete chloroplast genome (cpDNA) sequence of *Taxus yunnanensis* was determined from Illumina HiSeq pair-end sequencing data in this study. The cpDNA is 129,190 bp in length. Like other species of taxus genus, the chloroplast genome of *T. yunnanensis* has lost one of the large inverted repeats (IRs). The genome contains 116 genes, including 82 protein-coding genes, 4 ribosomal RNA genes, and 30 transfer RNA genes. Further phylogenomic analysis showed that *T. yunnanensis* closed to *T. brevifolia* in Lauraceae family.

*Taxus yunnanensis* is the species of the genus *Taxus* within the family Taxaceae, an evergreen tree commonly knwon as Hongdoushan, and grown mainly in Yunnan Province of China (Banskota et al. 2003; Ha et al. 2014). More than 550 taxane diterpenoids have been isolated from *Taxus* species (Wang et al. 2011). *Taxus yunnanensis* is considered as a promising source of taxane diterpenes, and more Taxol derivatives have biological activities such as antitumor, anticancer, anti-inflammatory, antiviral (Baloglu and Kingston 1999; Wang et al. 2011). Therefore, *T. yunnanensis* has huge medicinal value. However, there has been no genomic studies on *T. yunnanensis*.

Herein, we reported and characterized the complete *T. yunnanensis* plastid genome. The GenBank accession number is MT536348. One *T. yunnanensis* individual (specimen number: 2020013) was collected from Kunming, Yunnan Province of China (25°14 '28" N, 102°75 '43" E). The specimen is stored at Yunnan Academy of Forestry Herbarium, Kunming, China and the accession number is ZJFEP223. DNA was extracted from its fresh leaves using DNA Plantzol Reagent (Invitrogen, Carlsbad, CA, USA).

Paired-end reads were sequenced by using Illumina HiSeq system (Illumina, San Diego, CA). In total, about 20.2 million high-quality clean reads were generated with adaptors trimmed. Aligning, assembly, and annotation were conducted by CLC de novo assembler (CLC Bio, Aarhus, Denmark), BLAST, GeSeq (Tillich et al. 2017), and GENEIOUS v 11.0.5 (Biomatters Ltd, Auckland, New Zealand). To confirm the phylogenetic position of *T. yunnanensis*, other thirteen species of *Taxus* genus from NCBI were aligned using MAFFT v.7 (Katoh and Standley 2013). The Auto algorithm in the MAFFT alignment software was used to align the twelve complete genome sequences and the G-INS-i algorithm was used to align the partial complex sequences. The maximum likelihood (ML) bootstrap analysis was conducted using RAxML (Stamatakis 2006); bootstrap probability values were calculated from 1000 replicates. *Torreya fargesii* (KT027377) and *Torreya nucifera* (MK978775) were served as the out-group.

The complete *T. yunnanensis* plastid genome is a circular DNA molecule with the length of 129,190 bp, the overall GC content of the whole genome is 34.6%, like other species of taxus genus, the chloroplast genome of T. yunnanensis has lost one of the large inverted repeats (IRs). The plastid genome contained 116 genes, including 82 protein-coding genes, 4 ribosomal RNA genes, and 30 transfer RNA genes. Phylogenetic analysis showed that *T. yunnanensis* closed to *T. brevifolia* in *Taxus* genus (Figure 1). The determination of the complete plastid genome sequences provided new molecular data to illuminate the *Taxus* genus evolution.

**Figure 1.**
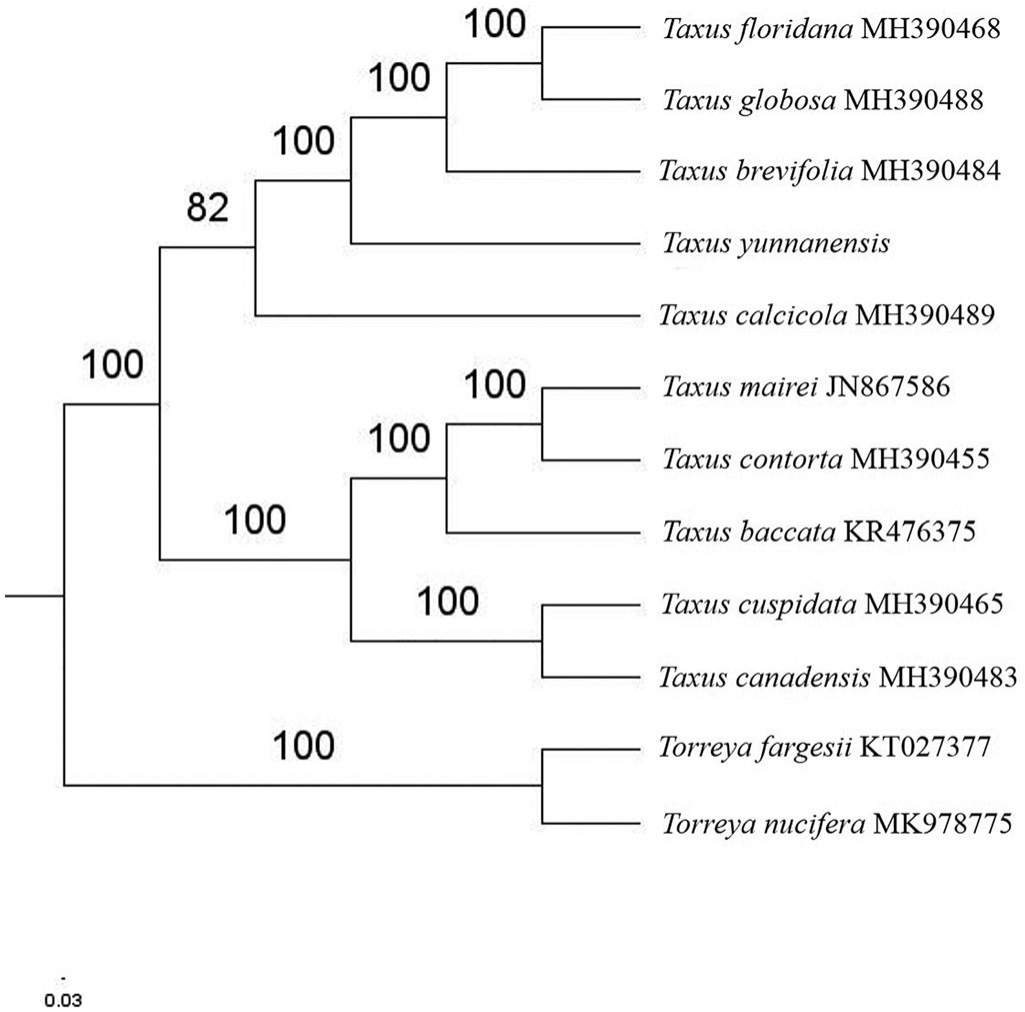
The maximum-likelihood tree based on the ten chloroplast genomes of *Taxus* genus. The bootstrap value based on 1000 replicates is shown on each node.

## Data Availability

The data that support the findings of this study are openly available in NCBI GenBank database at (https://www.ncbi.nlm.nih.gov) with the accession number is MT536348, which permits unrestricted use, distribution, and reproduction in any medium, provided the original work is properly cited.
